# Differential gene content and gene expression for bacterial evolution and speciation of *Shewanella* in terms of biosynthesis of heme and heme-requiring proteins

**DOI:** 10.1186/s12866-019-1549-9

**Published:** 2019-07-30

**Authors:** Jingcheng Dai, Yaqi Liu, Shuangyuan Liu, Shuyang Li, Na Gao, Jing Wang, Jizhong Zhou, Dongru Qiu

**Affiliations:** 1 Institute of Hydrobiology, Chinese Academy of Sciences, Wuhan, 430072 Hubei Province China; 2 University of Chinese Academy of Sciences, Beijing, 100049 China; 3 Institute for Environmental Genomics, and Department of Microbiology and Plant Biology, University of Oklahoma, Norman, OK 73019 USA; 4 Earth Science Division, Lawrence Berkeley National Laboratory, Berkeley, CA 94270 USA

**Keywords:** Ferrochelatase, Shewanella, Protoporphyrin IX, Cytochrome, Hemin

## Abstract

**Background:**

Most species of *Shewanella* harbor two ferrochelatase paralogues for the biosynthesis of *c*-type cytochromes, which are crucial for their respiratory versatility*.* In our previous study of the *Shewanella loihica* PV-4 strain, we found that the disruption of *hemH1* but not *hemH2* resulted in a significant accumulation of extracellular protoporphyrin IX (PPIX), but it is different in *Shewanella oneidensis* MR-1. Hence, the function and transcriptional regulation of two ferrochelatase genes, *hemH1* and *hemH2*, are investigated in *S. oneidensis* MR-1.

**Result:**

In the present study, deletion of either *hemH1* or *hemH2* in *S. oneidensis* MR-1 did not lead to overproduction of extracellular protoporphyrin IX (PPIX) as previously described in the *hemH1* mutants of *S. loihica* PV-4. Moreover, supplement of exogenous hemins made it possible to generate the *hemH1* and *hemH2* double mutant in MR-1, but not in PV-4. Under aerobic condition, exogenous hemins were required for the growth of MR-1Δ*hemH1*Δ*hemH2*, which also overproduced extracellular PPIX. These results suggest that heme is essential for aerobic growth of *Shewanella* species and MR-1 could also uptake hemin for biosynthesis of essential cytochrome(s) and respiration. Besides, the exogenous hemin mediated CymA cytochrome maturation and the cellular KatB catalase activity. Both *hemH* paralogues were transcribed in wild-type MR-1, and the *hemH2* transcription was remarkably up-regulated in MR-1Δ*hemH1* mutant to compensate for the loss of *hemH1*. The periplasmic glutathione peroxidase gene *pgpD*, located in the same operon with *hemH2*, and a large gene cluster coding for iron, heme (hemin) uptake systems are absent in the PV-4 genome.

**Conclusion:**

Our results indicate that the genetic divergence in gene content and gene expression between these *Shewanella* species, accounting for the phenotypic difference described here, might be due to their speciation and adaptation to the specific habitats (iron-rich deep-sea vent versus iron-poor freshwater) in which they evolved and the generated mutants could potentially be utilized for commercial production of PPIX.

**Electronic supplementary material:**

The online version of this article (10.1186/s12866-019-1549-9) contains supplementary material, which is available to authorized users.

## Background

The *Shewanella* species have been frequently isolated from redox-stratified environments and are renowned for their respiratory versatility and psychrophily [1, 2]. The respiratory versatility of *Shewanella* is mainly dependent on the ability to express a wide variety of different terminal electron acceptors and the multiple *c*-type cytochromes encoded in their genome, and high content of cytochromes usually confer an orange or pink color to cultures [1, 2]. It is intriguing how the synthesis of heme and expression of apocytochromes are balanced and regulated in these *Shewanella* strains. The heme biosynthesis pathway in *Shewanella* was reconstructed based on the KEGG pathway, genome sequencing and annotation, and it is demonstrated that the biosynthesis pathways for protoporphyrin IX (PPIX), heme, and *c*-type cytochromes are highly conserved between the Gamma-proteobacteria *Escherichia* and *Shewanella* as previously described [3, 4]. Unlike the model strain *Escherichia coli*, most *Shewanella* species harbor two ferrochelatase paralogues for heme synthesis. Abnormal accumulation of PPIX has been previously described in a *hemH* mutant of *Escherichia coli,* while the levels of PPIX accumulation was quite low [5–7]. For the high-level production of porphyrins, Assembly of highly standardized gene fragments in *E. coli* was conducted, and the state of the art PPIX-production levels was 50 mg/L [8].

As we all know, the *hemH* gene encodes the enzyme ferrochelatase, which catalyzes the last step of heme synthesis by inserting a ferrous ion (Fe^2+^) into the porphyrin ring of PPIX to form heme. Besides, the oxidized form (Fe^3+^) is known as hemin and is more soluble. However, both are commonly called “heme”. It also was reported that there are many different noncanonical pathways of heme biosynthesis in many different bacteria taxa such as *Firmicutes*, *Actinobacteria*, *Archaea*, sulfate-reducing bacteria and *Proteobacteria* [9–11]. As a prosthetic group in many proteins and enzymes, heme plays significant roles in many fundamental biological processes [12], such as respiration (heme-containing cytochromes), activation of O-O bond (heme peroxidase), O_2_ transport (hemoglobin) [13], gas sensing and signal transduction (nitric oxide synthase and CooA) [14], and control of gene expression [15]. Because heme can also serve as a source of both iron and porphyrin for microbes, most of the pathogenic bacteria such as *Pseudomonas aeruginosa*, *Neisseria meningitidis* and *Yersinia pestis* lack the heme biosynthetic pathway and requires exogenous heme for aerobic growth [16–20]. In *Staphylococcus aureus*, there are both of the exogenous heme acquisition and endogenous heme biosynthesis [21]. Rarely have non-pathogenic bacteria like MR-1 produce not only endogenous heme, but also assimilate the exogenous hemin. In addition, such a PPIX-overproducing phenotype has not been reported previously i*n S. oneidensis* MR-1 and had not been found in our large scale transposon mutagenesis in this strain [3], and deletion of *hemH1* in MR-1 did not result in a similar PPIX-overproducing phenotype.

To further explore the cellular functions of two HemH paralogues and increase the PPIX-production levels, we successfully generated the *hemH1* and *hemH2* double mutant in MR-1 with supplement of exogenous hemin for the first time and analyzed their expression in the wild type strains versus the single mutants. However, we were still unable to generate the *hemH1* and *hemH2* double mutant in PV-4 even with supplement of hemins. Moreover, the transcription and translation of some hemoproteins-coding genes were profiled, and the maturation and activation of these heme-containing proteins, such as CymA and KatB, were analyzed. Our results indicate that the observed phenotypic difference and speciation between PV-4 and MR-1 strains were probably due to the genetic divergence in terms of both gene content (presence or absence of heme/hemin uptake systems) and gene expression (the differential expression of *hemH* paralogues), meanwhile, the DNA engineering and traces of hemin for a desirable strain contribute to the high-level overproduction of extracellular PPIX.

## Results

### Deletion of either *hemH1* or *hemH2* did not result in obvious phenotypic changes in MR-1

**Fig. 1:**
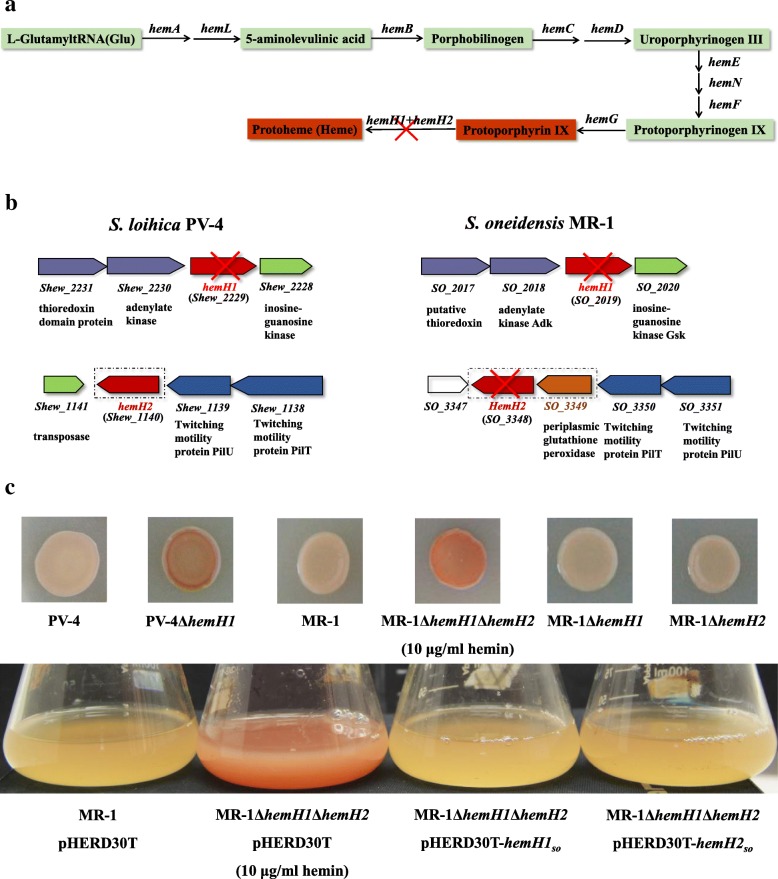
Effects of *hemH1* and *hemH2* double deletions on phenotypes of *S. oneidensis* MR-1. **a** The heme biosynthesis pathway; **b** Organization of the ferrochelatase genes *hemH1* and *hemH2* in *S. loihica* PV-4 and *S. oneidensis* MR-1. The operon encodes a periplasmic glutathione peroxidase (SO_3349) and a ferrochelatase paralogue (*hemH2*) in strain MR-1 while the former is absent in strain PV-4; **c** Inactivation of both ferrochelatase genes (*hemH1* and *hemH2*) resulted in overproduction of protoporphyrin IX in MR-1. Cell colonies grown from a droplet of mid-log-phase culture (OD_600_ of ~ 0.2) for each indicated strain on LB plates (supplemented with 10 μg/ml of hemin), the *hemH1* and *hemH2* double mutant exhibited a red-color phenotype while deletion of either *hemH1* or *hemH2* did not cause the same phenotypic change. In the genetic complementation analyses on the PPIX-overproducing *hemH1* deletion mutant PV-4**Δ***hemH1*, the plasmid-borne wild-type *hemH1* or *hemH2* gene restored the phenotype of the mutant to the same pink color of the wild-type strain carrying the empty vector

PPIX is a precursor for heme synthesized in the highly conserved heme biosynthesis pathway [22, 23]. All of the genes involved in the pathway, shown schematically in Fig. 1a, have been found in the sequenced *S. loihica* PV-4 and *S. oneidensis* MR-1 genomes. These genes are distributed at distinct genomic loci, and extraordinarily there are two paralogous *hemH* genes, *hemH1* and *hemH2*, whose genomic contexts are shown in Fig. 1b. Interestingly, there is a periplasmic glutathione peroxidase gene *pgpD* located in the same operon with *hemH2* in MR-1 but not in PV-4. Actually, there are two ferrochelatase paralogues in the genome of most *Shewanella* strains. The in-frame deletion of *hemH1* but not *hemH2* in PV-4 strain led to overproduction of red-colored PPIX (Fig. 1c) [4]. Meanwhile, we also had conducted large scales of transposon mutagenesis in the strain MR-1, a total of over 20,000 mutants were obtained with covering approximately 4 genome equivalents, but we had never isolated the PPIX-releasing red-colored mutants. Therefore, we also generated Δ*hemH1* or Δ*hemH2 single in-frame deletion mutant in MR-1, h*owever, deletion of either *hemH1* or *hemH2* did not lead to the red-colored colony phenotype in the MR-1 mutants (Fig. 1c). Particularly, unlike the *Escherichia coli* containing only one ferrochelatase gene which could be deleted [24], the two ferrochelatase *hemH* paralogs of either PV-4 or MR-1 strain could not be deleted to generate the *hemH1* and *hemH2* double mutant. These results indicate that HemH1 and HemH2 were functionally redundant and deletion of each of them did not significantly affect the homeostasis of heme and biosynthesis of *c*-type cytochromes in MR-1. These results prompted us to explore such genetic divergence regarding regulatory mechanism underlying in biosynthesis pathway of PPIX, heme and *c*-type cytochromes between the PV-4 and MR-1 species with remarkable difference.

### *S. oneidensis* MR-1 *hemH1* and *hemH2* double mutant is auxotrophic for hemin

**Fig. 2:**
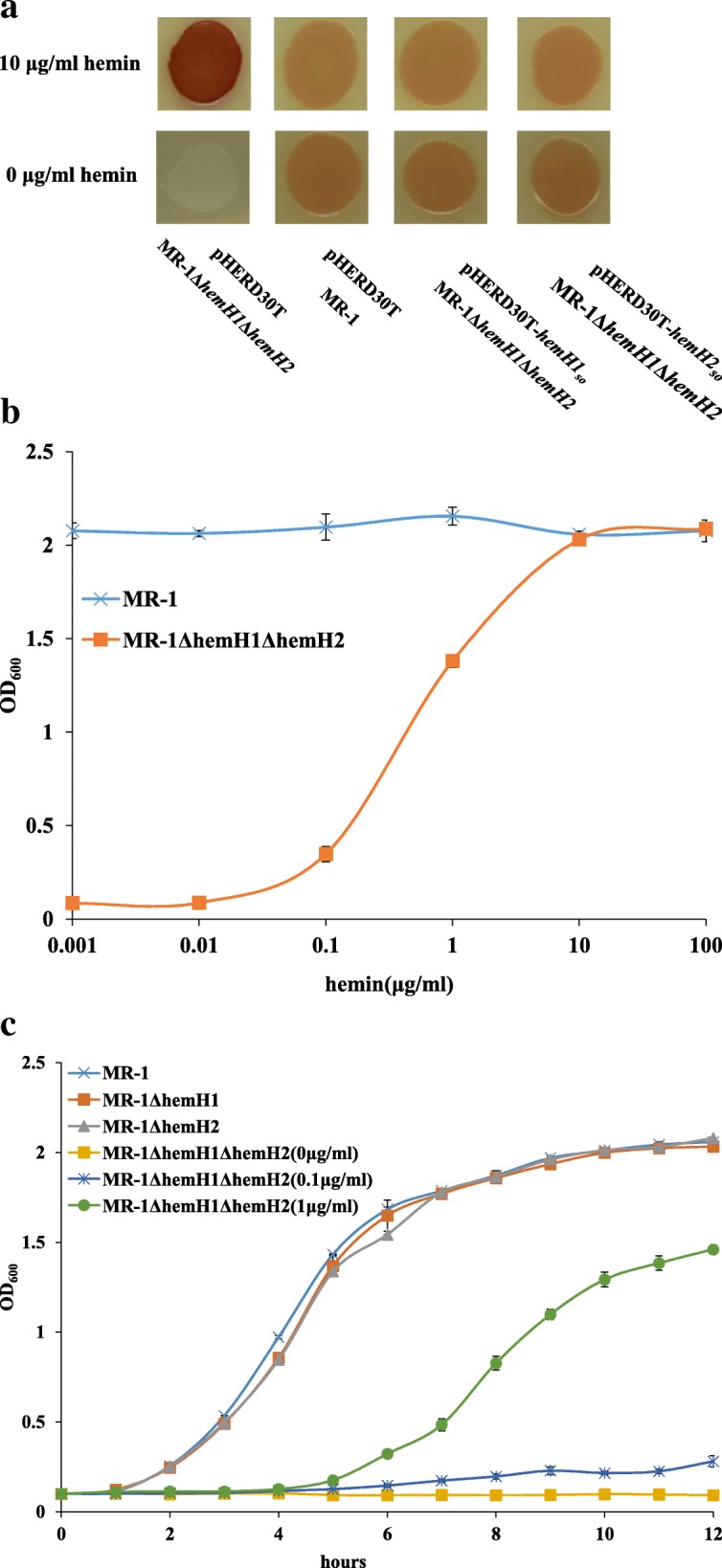
Effects of supplemented hemin concentrations on the bacterial growth of the *hemH1* and *hemH2* double mutant. **a** The *hemH1* and *hemH2* double mutant exhibited red-colored phenotype with supplement of hemin (10 μg/ml) while the double mutant could not grow without supplement of hemin. In the genetic complementation analyses, the plasmid borne wild-type *hemH1* or *hemH2* gene restored phenotype of the *hemH1* and *hemH2* double mutant to that of the wild-type strain carrying the empty vector with or without supplement of hemin; **b** The bacterial strains were grown in the LB broth containing 0, 0.001, 0.01, 0.1, 1, 10, 100 μg/ml of hemin and 15 μg /ml of gentamycin, and incubated at 28 °C for 18 h; **c** The growth of the MR-1, MR-1Δ*hemH1*, MR-1Δ*hemH2*, MR-1Δ*hemH1*Δ*hemH2* in the LB broth with 0, 0.1, 1 μg/ml of hemin over time was measured. Growth was determined by measuring OD_600_ values

Previously we had been unable to generate the double mutant of *hemH1* and *hemH2* in either PV-4 or MR-1 strain, indicating that heme is probably essential for aerobic respiration of *Shewanella* species. Therefore, we supplemented hemin (the oxidized form (Fe^3+^) *with higher solubility than heme*) to bacterial culture media, and we could successfully generate the *hemH1* and *hemH2* double mutant in the MR-1 strain under this condition (Additional file 1: Figure S1). The double mutant could not grow without supplement of hemin, but the cell growth of single mutants was not affected as compared with that of the wild-type MR-1 strain (Fig. 1c and Fig. 2a). Moreover, the cell growth of the double mutant was dependent on the dosage of added hemin and gradually caught up with that of the wild-type strain with increasing dosage of hemin supplement (Fig. 2b and c). Therefore, it is clear that heme deficiency may affect the synthesis of hemoproteins required for bacterial growth rate of MR-1Δ*hemH1*Δ*hemH2* cells. The double mutant achieved a cell density (optical density at 600 nm) similar to that of the wild-type MR-1 strain when the hemin supplement was increased up to the concentration of 10 μg /ml.

To further confirm the functional redundancy between HemH1 and HemH2 in MR-1, the expression of wild-type *hemH1*_*so*_ or *hemH2*_*so*_ gene *in trans* in the MR-1Δ*hemH1*Δ*hemH2* mutant fully reversed the red-colored phenotype, meanwhile, rescued the growth of MR-1Δ*hemH1*Δ*hemH2* mutant without hemin supplement (Fig. 1c and Fig. 2a). However, the *hemH1* and *hemH2* double mutant still could not be generated in the PV-4 strain, probably due to the absence of MR-1-harbored hemin uptake system in PV-4 (Fig. 7a). It is suggested that the *hemH1* and *hemH2* double mutant of MR-1 could uptake the added hemin for *c*-type cytochrome biosynthesis in the absence of HemH1 and HemH2.

### Chemical analyses of the extracellular compound in MR-1Δ***hemH1***Δ*hemH2*

**Fig. 3:**
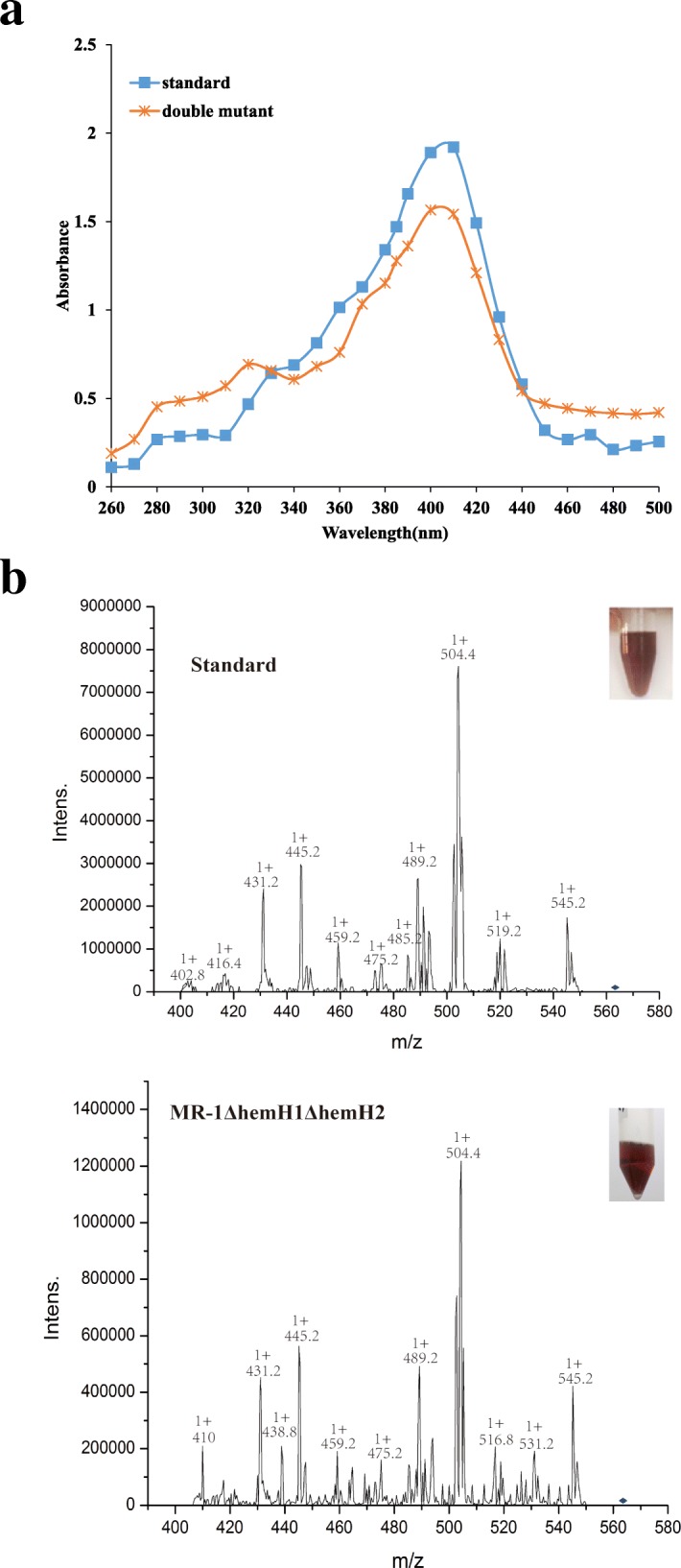
The chemical analyses of the extracellular compound. The samples and a PPIX standard (Sigma-Aldrich, St. Louis, MO) were dissolved in a solution containing 90% acetone and 10% 0.1 M NH_4_OH. **a** Electrospray ionization tandem mass spectrometry (ESI-MS/MS, precursor ion: 563.2-PPIX) analyses showing almost identical structure of the PPIX standard and the bacterial samples; **b** Ultraviolet-visible spectrograms of the bacterial extract (the *hemH1* and *hemH2* double mutant) and PPIX standard. The absorbance was measured at every 10 nm (nm) with a spectrometer (UV-1800, Mapada) and quartz cuvettes

In our previous research, the red pigments of PPIX gradually accumulated, conferring a deep red color to the colonies or cell culture broth of PV-4*ΔhemH1* mutant (Fig. 1c) [4]. However, MR-1Δ*hemH1* exhibited no PPIX-overproducing phenotype, and this obvious red color had not been described in the *hemH* mutant of *E. coli* [24, 25]. Interestingly, the double deletion of *hemH1* and *hemH2* in MR-1 strain led to overproduction of red-colored substance. It was clear that the red pigment was secreted or leaked into the media by the mutants because they accumulated into aggregates floating in the culture broth, therefore, the extracellular red pigments could be easily extracted and purified. To determine whether the red pigment was PPIX, a series of spectrometric analyses were conducted to determine the structure of this bacterial product [26]. The ultraviolet-visible absorbance spectra of the *hemH1* and *hemH2* double mutant extract and the commercially available PPIX standard was measured and compared. Their spectrograms were very similar and the maximum absorbance of both PPIX standard and the mutant extract occurred at around 405 nm (Fig. 3a). However, the wild-type (WT) MR-1 strain extract did not generate an absorbance peak at about 405 nm, indicating the red-colored compound was PPIX as expected (Additional file 1: Figure S2), and there was no PPIX accumulated in WT. Further structural analyses were conducted by using electrospray ionization-tandem mass spectrometry (ESI-MS/MS) (Fig. 3b), showing that the fragment profile of the double mutant sample was very similar to that of the commercial PPIX standard. Taken together, these results demonstrated that the red extracellular compound in the *hemH1* and *hemH2* double mutant of MR-1 was indeed PPIX. More importantly, the fermenter cultivation showed that the yield of the MR-1Δ*hemH1*Δ*hemH2* mutant was about 200–400% higher than those of PV-4Δ*hemH1* single mutant that we previously generated (US patent No. WO2014144329 A2).

### Different transcriptional patterns of *hemH* paralogues in *shewanella*

**Fig. 4:**
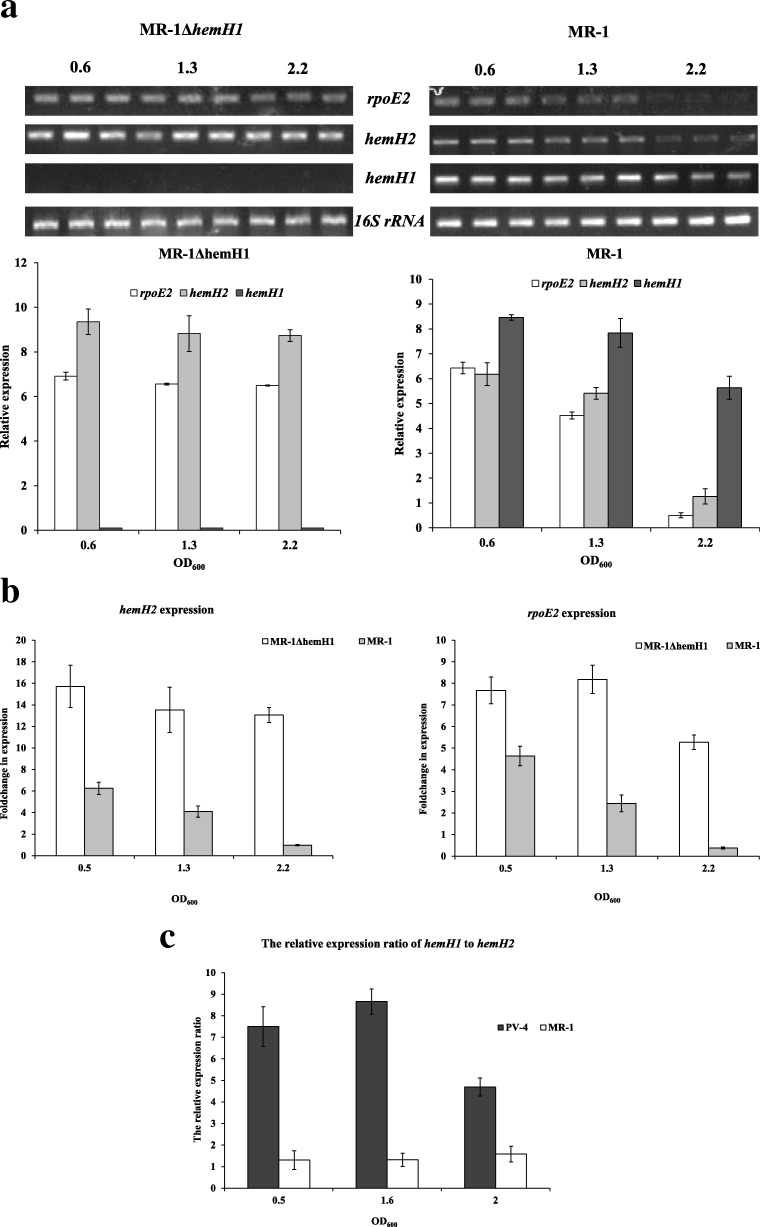
Transcriptional analyses of *rpoE2* and *hemH* paralogues in the wild-type strain and the *hemH1*-null mutant of *S. oneidensis* MR-1. **a** Semi-quantitative RT-PCR analyses of *rpoE2* and *hemH2* transcripts in MR-1 and MR-1Δ*hemH1* strains; **b** The Real-time PCR analyses of *hemH2* transcripts in MR-1 and MR-1Δ*hemH1* strains; **c** the relative expression ratio of *hemH1* to *hemH2* in PV-4 and MR-1. Cell samples of the strains were collected for RNA extraction at optical density (OD_600_) of 0.6, 1.3, and 2.2. Transcription of the *16S rRNA* genes was analyzed and used as the internal control gene. The assays were performed in triplicates and the error bars represented the standard deviation (SD) of triplicate independent samples

We have found there are two ferrochelatase paralogues in the most *Shewanella* strains. Based on *S. oneidensis* MR-1 and *S. loihica* PV-4, we have monitored the temporal expression patterns of *hemH1* and *hemH2* with RT-PCR and qRT-PCR analyses. In wild-type MR-1 strain, the two *hemH* paralogues and *rpoE2* were nearly equivalently transcribed and the transcription of *rpoE2* and *hemH2* was remarkably up-regulated in the *hemH1* mutants of MR-1 (Fig. 4a and b). These results suggest that the enhanced expression of *hemH2* was capable of compensating for the loss of *hemH1* and maintain the ferrochelatase activity for heme biosynthesis. Therefore, no PPIX was overproduced in the MR-1Δ*hemH1* mutant. Interestingly, we have previously demonstrated that the expression level of *hemH2* was too low to be detectable in PV-4 strain [4]. To further understand the differential transcriptional regulation of *hemH* paralogues between the MR-1 and PV-4 strains, the qRT-PCR analyses were conducted and it was shown that the relative expression ratio of *hemH1* to *hemH2* in PV-4 was much higher than that in MR-1 (Fig. 4c), which partly explains why PV-4Δ*hemH1* but not MR-1Δ*hemH1* exhibited PPIX-accumulation phenotype.

### Exogenous hemins did not restore the resistance of MR-1Δ***hemH1***Δ*hemH2* to oxidative stress and light

**Fig. 5:**
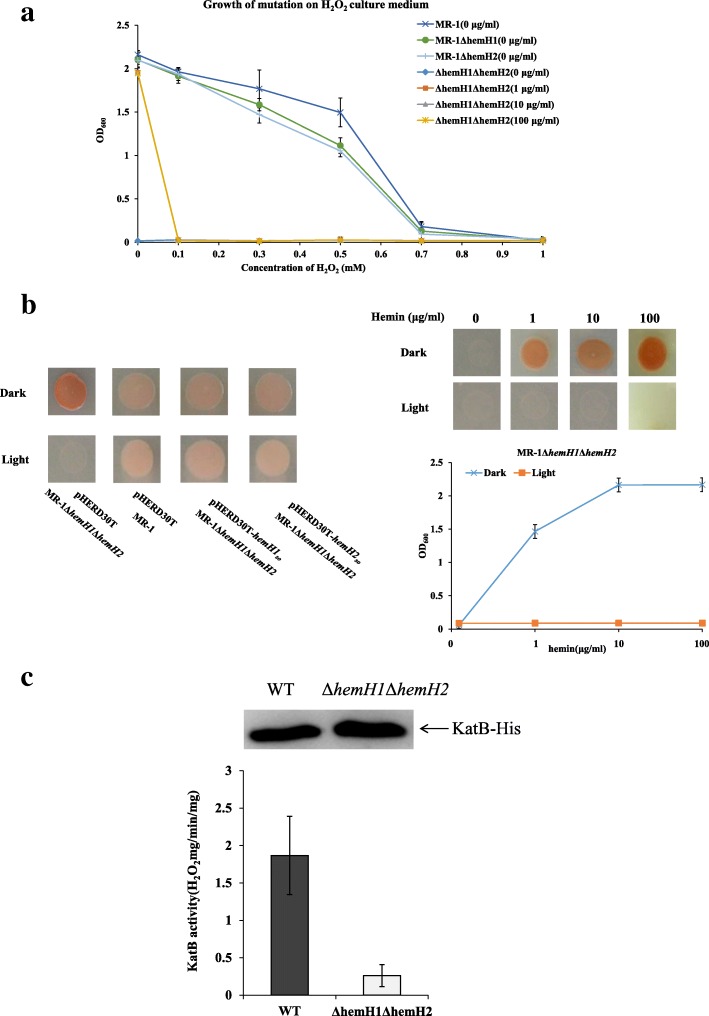
Effects of visible light and hydrogen peroxide (H_2_O_2_) on the MR-1Δ*hemH1*Δ*hemH2* mutant. **a** Effects of hydrogen peroxide (H_2_O_2_) addition on the growth of the MR-1Δ*hemH1*Δ*hemH2* mutant. The MR-1 wild type strain, the *hemH1*, the *hemH2* in-frame deletion mutants and the *hemH1-hemH2* double mutant strain were grown in LB broth containing 0, 0.1, 0.3, 0.5, 0.7 and 1 mM of hydrogen peroxide and incubated at 28 °C for 18 h. Different concentrations (0, 0.1, 1, 10 μg/ml) of hemin didn’t rescue the growth rate of the hemH1-hemH2 double mutant, when these strains were grown in the LB broth containing 0, 0.1, 0.3, 0.5, 0.7 and 1 mM of hydrogen peroxide and incubated at 28 °C for 18 h; **b** Effects of visible light on the growth of the MR-1Δ*hemH1*Δ*hemH2* mutant. Cell colonies grown from a droplet of mid-log-phase culture (OD_600_ of ~ 0.2) for each indicated strain on LB plates (supplemented with 10 μg/ml of hemin and 15 μg/ml gentamycin). Experiments were conducted under visible light (about 700~1,000 lx) and dark condition on LB plates. The wild type MR-1 carrying the empty vector was used as control, the double deletion mutant MR-1Δ*hemH1*Δ*hemH2* contained the empty vector, pHERD30T-*hemH1* and pHERD30T-*hemH2*, respectively, were compared. Different concentrations of supplement hemin failed to rescue the phenotype and the growth rate of the *hemH1-hemH2* double mutant, when these strains were grown in the LB broth containing 0, 0.1, 1, 10, 100 μg/ml of hemin and incubated at 28 °C for 18 h; **c** The KatB-dependent peroxidase activity assays of MR-1 and the double mutant MR-1Δ*hemH1*Δ*hemH2*

Light-exposure of PPIX as a photosensitizer is known to result in a high-energy state (PPIX^+^), which could interact with molecular oxygen to generate the highly reactive oxygen species (ROS) that cause cell damage and death [27, 28]. Many bacteria employ catalases and peroxidases to keep the steady-state level of ROS below the threshold of toxicity [29, 30]. Though deletion of either *hemH1* or *hemH2* in MR-1 did not affect the bacterial resistance to hydrogen peroxide, the double mutant became very sensitive to the oxidative stress (Fig. 5a). Interestingly, it was revealed that under light irradiance the bacterial growth of the *hemH* double mutant could not be recovered by addition of the same level of 10 μg/ml hemin while under dark condition the double mutant, as expected, was viable and exhibited the red-colored colony phenotype (Fig. 5b). These results are not consistent with our previous findings that visible light *did not cause the lack of cell growth* of PV-4Δ*hemH1mutant although* the PPIX-overproducing red-colored phenotype could be reversed*.* Though the double mutant grown in a medium supplemented with 100 μg/ml hemin was not viable under light exposure (Fig. 5b), expression of either *hemH1* or *hemH2 in trans* completely rescued the cell growth of the double mutant under light exposure. Meanwhile, the bacterial resistance to hydrogen peroxide stress had not been recovered by addition of high levels of exogenous hemins up to 100 μg/ml (Fig. 5a). These results are inconsistent with the previous reports that hemin could quench high-energy states and minimize photolysis [31, 32]*.* When the *katB* gene encoding a heme-requiring catalase/peroxidase, was overexpressed in both of the wild-type MR-1 strain and the double mutant, the KatB protein titers of MR-1Δ*hemH1*Δ*hemH2* were similar to those of WT (Fig. 5c). However, the KatB-dependent peroxidase activity declined by about 7-folds, suggesting that the maturation and activation of KatB catalase is dependent on heme availability.

### Nitrate reduction could not restored by increasing supplement of hemins

**Fig. 6:**
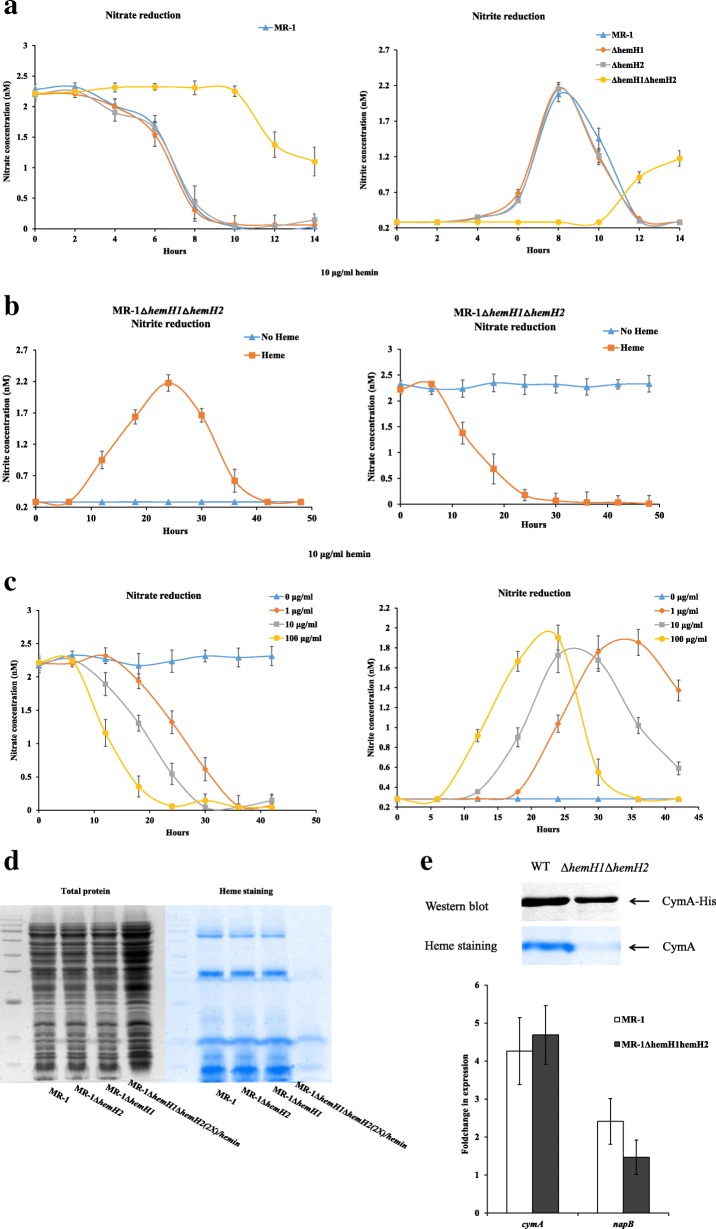
Effects of *hemH1* and *hemH2* double deletions on *c*-type cytochrome synthesis and nitrate reduction in *S. oneidensis* MR-1. **a** Bacteria were cultivated in the modified M1 minimal media supplemented with 2 mM sodium nitrate under microoxic conditions (in tightly capped tubes incubated without shaking). The blank represents the culture media without bacterial inoculation. Error bars represent SD; **b** Hemin could rescue the nitrate reduction capacity of MR-1Δ*hemH1*Δ*hemH2* under microoxic conditions; **c** The effect of different hemin concentration(0, 0.1, 1, 10 μg/ml) on nitration reduction in *hemH1-hemH2* double mutant strain MR-1Δ*hemH1*Δ*hemH2* under microoxic conditions; **d** Effects of *hemH1-hemH2* double deletion on *c*-type cytochrome synthesis in MR-1. Total protein (left) content and Heme staining (right) analyses of *c*-type cytochromes in the following stains: MR-1 wild-type, MR-1Δ*hemH1*, MR-1Δ*hemH2*, MR-1Δ*hemH1*Δ*hemH2* (2X means the double quantity of sample). After cell disruption, the supernatants containing the cellular protein fraction were resuspended in the SDS loading buffer and then incubated at 37 °C for 1 h; **e** Top panel: The CymA protein content of the wild-type strain MR-1 cells is ∼2-fold higher than that of the double mutant MR-1Δ*hemH1*Δ*hemH2* cells, but the heme-staining analyses showed that the CymA protein were not detected. Cells were grown in M1 medium supplemented with 2 mM sodium nitrate and 0.1 μg/ml hemin under microoxic conditions, and CymA protein content was quantified by using western blotting and densitometry. The lanes contained equivalent total proteins. Bottom panel: The Real-time PCR analyses of *napB* and *cymA* transcripts in MR-1 and MR-1Δ*hemH1hemH2* strains

Since the *hemH1* and *hemH2* double mutant could not grow aerobically, the anaerobic respiration was also tested. Deletion of either *hemH1* or *hemH2* in MR-1 did not affect the nitrate reduction, but there was significant influence on the nitrate reduction rates of the double mutant (Fig. 6a). As described above, the cell growth of the double mutant could catch up with that of the wild-type strain when the exogenous hemin increased up to 10 μg/ml (Fig. 2b). Similarly, with increased supplement of hemins up to 100 μg/ml, the nitrate reduction rates of the double mutant were gradually enhanced, although the rates were still not comparable to those of the wild type strain (Fig. 6b and c). The nitrate reduction process usually takes about 8–10 h in MR-1 under our test conditions, while it took 25–30 h for MR-1Δ*hemH1*Δ*hemH2* to reduce nitrate even supplemented with up to 100 μg/ml of exogenous hemins (Fig. 6a and c).

In order to determine whether MR-1Δ*hemH1*Δ*hemH2* cells are inefficient at producing heme-requiring proteins even if exogenous hemins were provided in sufficient amounts, heme-staining analyses of the cellular proteins were carried out. The *c*-type cytochrome levels of the double mutant MR-1Δ*hemH1*Δ*hemH2* were significantly lower while the total protein content were twice as much as that of MR-1 (Fig. 6d). The other important heme-requiring proteins in MR-1 were listed in the Additional file 1: Table S1. Two *c*-type cytochromes, NapB and CymA, are part of periplasmic nitrate reductase Nap in MR-1. Our results are consistent with previous findings that the tetraheme cytochrome CymA is essential for nitrate reduction in both MR-1 and *S. putrefaciens* W3–18-1 strains [33]. To further explore the biosynthesis of these two *c*-type cytochromes under supplement of exogenous hemins, we found that there was no significant difference in the transcription levels of *cymA* and *napB* genes between the wild-type MR-1 and the MR-1Δ*hemH1*Δ*hemH2* double mutant (Fig. 6e). Although the *cymA* was overexpressed in the MR-1Δ*hemH1*Δ*hemH2* double mutant and wild-type strain cells for high levels of CymA apoproteins, there was significant difference in synthesis of *c-*type cytochromes observed between the double mutant and the wild-type MR-1 strain (Fig. 6e).

Taken together, it is shown that the exogenous hemins could be not readily available for synthesis of *c*-type cytochromes and temporal heme deficiency may have affected the maturation of the CymA and other cytochromes. The externally supplemented soluble hemin (PPIX-Fe^3+^ chloride) is an oxidized form of heme, which may not be readily utilized for biosynthesis of the heme-requiring proteins such as catalase, peroxidase, nitrate and nitrite reductases. The post-translational maturation of heme-containing proteins may take a long time without enough endogenous heme supply.

### Putative heme/hemin transport system is present in *S. oneidensis* MR-1 but not in *S. loihica* PV-4

**Fig. 7:**
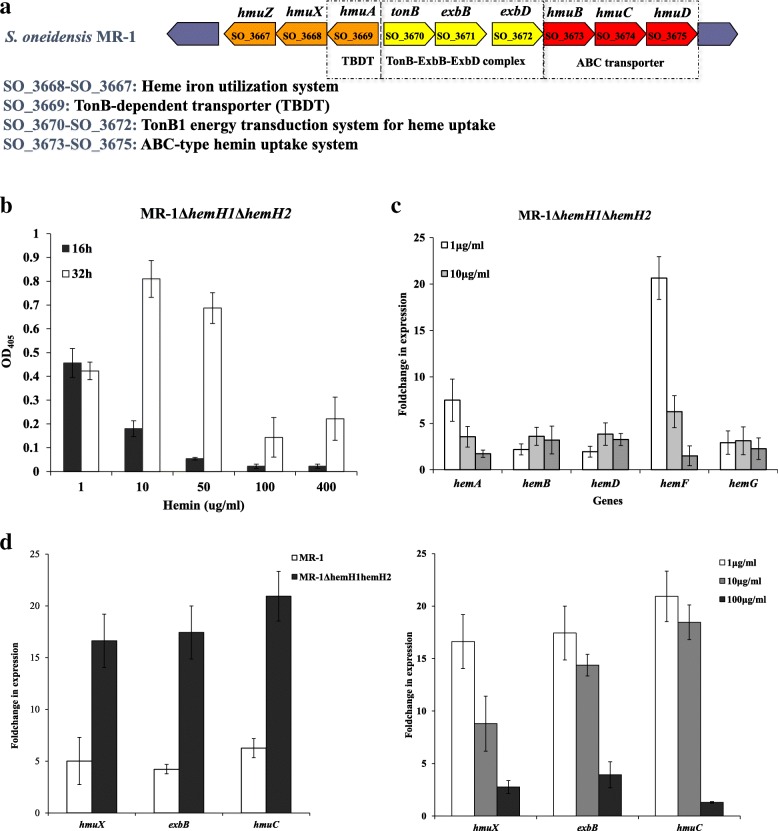
The uptake of exogenous hemin depends on TonB1 energy transduction system in *S. oneidensis* MR-1. **a** Organization of the putative hemin uptake gene cluster in MR-1. The region corresponds to the open reading frames from SO_3667 to SO_3675 (left to right); **b** Effects of exogenous hemin supplement on the PPIX production of MR-1Δ*hemH1*Δ*hemH2*. Ultraviolet-visible spectrograms of the bacterial extract (the *hemH1* and *hemH2* double mutant). The absorbance was measured at a wavelength 405 nm with a spectrometer (UV-1800, Mapada) and quartz cuvettes; **c** Effects of exogenous hemin supplement on the transcription of *hemA*, *hemB*, *hemD*, *hemF*, *hemG* genes in the double mutant MR-1Δ*hemH1*Δ*hemH2*; **d** Transcriptional analyses of *hmuX, exbB* and *hmuC* in the wild-type strain and the *hemH1-hemH2* double mutants of MR-1. Effect of exogenous hemin supplement (0.1 μg/ml, 5 μg/ml, 40 μg/ml) on the transcription of *hmuX, exbB* and *hmuC* in the *hemH1-hemH2* double mutants of MR-1

We had been unable to generate a Δ*hemH1*Δ*hemH2* double mutant in PV-4 even with supplement of hemin, whereas we could obtain the Δ*hemH1*Δ*hemH2* double mutant in MR-1. We predicted that the difference might be due to the difference in heme uptake systems between PV-4 and MR-1. Although heme uptake mechanisms of *S. oneidensis* MR-1 have not been characterized to date, the genes coding for several putative heme/hemin transport are present in the genome (Fig. 7a), suggesting that *S. oneidensis* MR-1 might potentially assimilate exogenously supplied hemin. On the contrary, we found the putative heme/hemin transport system was absent in PV-4 by compared the genes function of MR-1 to that of PV-4 (Additional file 1: Table S2). More interestingly, the overproduction of PPIX was also suppressed in MR-1Δ*hemH1*Δ*hemH2* when the higher level of hemins was supplemented (Fig. 7b). These results indicated that the absorbed heme could also exert a feedback regulation on PPIX biosynthesis via a yet unknown mechanism. Therefore, we next explored the mechanism responsible for the suppression of PPIX synthesis upon hemin dosage. We confirmed that the *hemA* and *hemF* genes were significantly induced when 1–10 μg/ml of hemins were supplemented, whereas the other genes related to PPIX synthesis remained at basal levels (Fig. 7c). HemA (glutamyl-tRNA reductase) catalyzes the first step in heme synthesis (Fig. 1a), and this reaction is rate-limiting for pathway flux [34]. The prior studies showed that HemF and HemH were two heme biosynthetic enzymes induced by OxyR [4, 29]. Therefore, it was likely that OxyR activated the upregulation of *hemF* by the PPIX accumulation-induced oxidative stress.

Moreover, we also confirmed that *hmuX*, *humC* and *exbB* genes involved in hemin uptake were upregulated in MR-1Δ*hemH1*Δ*hemH2* as compared to that of MR-1, especially at the lower hemin concentrations tested (1 and 10 μg /ml) (Fig. 7d). It is well known that iron is an essential element for almost all organisms, including bacteria, and sophisticated mechanisms have evolved for sequestration of iron. One of which have been identified mostly in bacterial pathogens is to take up iron from free heme, for the uptake of iron from the environment [35]. Our research showed the *S. oneidensis* MR-1, like bacterial pathogens, has a well-characterized system, encoding the TonB-dependent heme iron sequestration and utilization system (*hmu*) and ABC-type hemin transporters (loci SO_3668-SO_3675 in MR-1) (Fig. 7a), to acquire exogenous heme. However, these genes are absent in the genome of PV-4.

## Discussion

*Shewanella* species are renowned for their respiratory flexibility, which is largely due to multiple *c*-type cytochromes [36]. Some other biologically important hemoproteins such as catalases and peroxidases, which display a wide range of functionalities, require sufficient heme supply [15]. Therefore, heme homeostasis is not only very crucial for cellular respiration but also critical for the basic processes of cellular life activities. Our purpose was to decipher the molecular basis underlying the PPIX biosynthesis and transcriptional regulation in response to heme imbalance and to lead to a better understanding of how different *Shewanella* species evolved and diverged under changing environmental conditions.

We could only generate the *hemH1* and *hemH2* double mutant in the MR-1 strain with supplement of hemins but not in PV-4 strain, which could not uptake and utilize external heme molecules in the absence of relevant genes (Fig. 7a). As expected, the double *hemH* mutant of MR-1 could overproduce PPIX as did the *hemH1* single mutants of PV-4. However, deletion of *hemH1* did not lead to overproduction of PPIX in the model strain *S. oneidensis* MR-1. The expression levels of another ferrochelatase paralogue, *hemH2,* were relatively low, but its transcription was remarkably up-regulated when *hemH1* was inactivated, indicating that the upregulated expression of *hemH2* could compensate for the loss of *hemH1* (Fig. 4a and b)*.* However, the expression of *hemH2* was too low in *S. loihica* PV-4 and the up-regulation of *hemH2* transcription was still not high enough for heme and *c*-type cytochrome biosynthesis in the *hemH1* mutants (Fig. 4c), resulting in PPIX release [4]. Our previous research suggested that the pgpD-hemH2 operon was regulated by the RpoE2 sigma factor involved in oxidative stress response in MR-1 strain (Dai et al. 2015), but the PV-4 strain harbor only the single gene operon of *hemH2*(Fig. 1b). We also demonstrated that the relative expression ratio of *hemH2* to *hemH1* in MR-1 was much higher than those of PV-4 (Fig. 4c). Therefore, we predicted that the up-regulated transcription of both *hemH2* and *rpoE2* in the *hemH1* deletion mutant of MR-1 strain might be dependent on activation of RpoE2 by the PPIX accumulation-induced oxidative stress, which was sensed by the the cognate anti-sigma factor ChrR. The presence of two functionally redundant HemH paralogues, whose expression is regulated in different manners, could maintain cellular homeostasis and well-balanced heme and c-type cytochrome biosynthesis pathways in the *Shewanella* strains with a large amount and a variety of c-type cytochromes. Taken together, one of the most remarkable differences between the PV-4Δ*hemH1* and MR-1Δ*hemH1* strains was the significant different expression levels of RpoE2-dependent *hemH2* orthologue, which accounts for the release of PPIX by PV-4Δ*hemH1*. The RpoE2-dependent promoter activity of *hemH2* gene of MR-1 might be stronger than that of PV-4 and ClustalW2 alignment showed that RpoE2-dependent promoter motifs in upstream of *hemH2* did have minor differences between the two *Shewanella* strains (Additional file 1: Figure S3). In other *Shewanella* strains, including *S. putrefaciens* W3–18-1 and CN-32, the two ferrochelatase paralogues may also be fully functional in terms of heme and cytochrome biosynthesis because we had not obtained the PPIX-overproducing mutants in our large-scale transposon mutagenesis (data not shown).

**Fig. 8:**
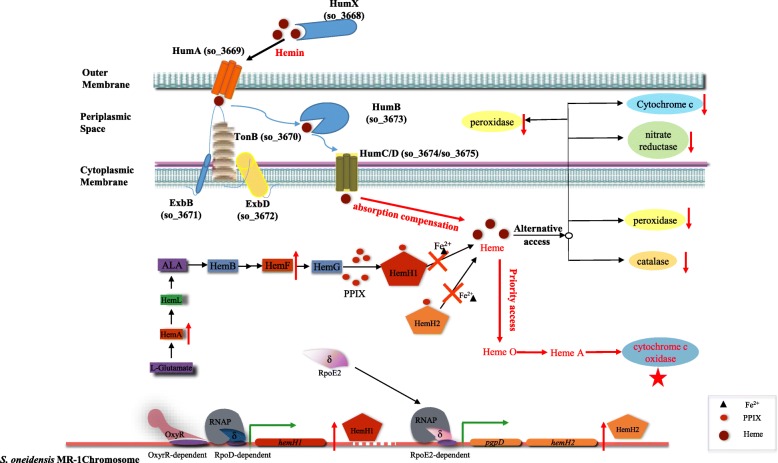
Schematic diagram illustrating the uptake pathway of exogenous hemin and cellular function and the access to cytochrome protein assemble in *S. oneidensis* MR-1 strain. Only the supplement of exogenous hemin made it possible to delete both *hemH1* and *hemH2* genes simultaneously in MR-1. The MR-1 strain could uptake exogenous hemin to compensate for loss of endogenous heme synthesis via the hemin uptake system. The hemin was transported into the cells and was mainly utilized to synthesize the respiration-related hemoproteins, especially the aerobic respiration-related proteins cytochrome *c* oxidase prior to other heme-requiring proteins such as catalase, peroxidase, and nitrate reductase. The upward red arrow indicates the upregulation of gene expression, the downward red arrow indicates the decrease of enzyme activity

Furthermore, we found that the increased supplement of hemins could not only recover the aerobic growth of the *hemH1* and *hemH2* double mutant, but also suppress the overproduction of PPIX as described above (Fig. 2b and Fig. 7b). Therefore, exogenous hemin supplement could facilitate the aerobic respiration of MR-1Δ*hemH1*Δ*hemH2.* In the *Lactobacillus casei* N87 strain, the analysis of gene expression suggested that exogenous hemin induced the transcription of *pox* and *cydABCD* genes [37]. The aerobic pathway for energy production involves pyruvate oxidase (POX) and the CydABCD is required for synthesis of functional cytochrome *bd* oxidase as a terminal O_2_ reductase in respiratory chain [38]. These results are consistent with our aerobic respiration tests. Unfortunately, hemin supply could not completely restore the rates of nitrate and nitrite reduction, which did not start as usual. There is a time lag in cell growth between the wild-type MR-1 and the double mutant under anaerobic condition, even though the concentration of exogenous hemin was added up to 100 μg/ml, ten times higher than those required for the normal level of aerobic cell growth. Heme is also utilized to defend cells against active species of oxygen, and could directly quenches ^1^O_2_ or impedes its generation by quenching excited states of PPIX [32, 39]. However, there was essentially no growth at any hemin supplement concentrations (0–100 μg/ml) when the mutant MR-1Δ*hemH1*Δ*hemH2* is in the light. Interestingly, we found that the expression of *cymA* and *napB,* like *katB,* exhibited no difference between the wild-type MR-1 and the double mutant MR-1Δ*hemH1*Δ*hemH2.* However, the KatB-dependent peroxidase activity and CymA cytochrome maturation in MR-1Δ*hemH1*Δ*hemH2* supplemented with 10 μg/ml hemin were significantly affected as compared to those of MR-1. These results are consistent with our anaerobic respiration and oxidative stress test results. Therefore, we concluded that the heme-mediated maturation and activation of hemoproteins may be affected though the gene transcription and apoprotein translation remained unaffected when heme synthesis is blocked and MR-1Δ*hemH1*Δ*hemH2* can only assimilate and utilize exogenously supplied hemin. Surprisingly, *Escherichia coli* can grow without heme, both in broth and in minimal media that contain fermentable sugars, since this bacterium has a set of glycolytic enzymes that produce ATP independently of respiration [39], while heme is required for respiration of *Shewanella*. We propose a model to depict the way to transport and utilization of exogenous hemins resources (Fig. 8). The bacterial cell could absorb exogenous hemin to compensate for lack of endogenous heme synthesis through the hemin uptake system, by which the hemin was transported into the cell and was mainly utilized to synthesize the respiration-related hemoproteins, particularly the aerobic respiration-related proteins such as cytochrome *c* oxidase, before other heme-requiring proteins such as catalase, peroxidase, and sulfite reductase were synthesized.

The closely related MR-1 and the PV-4 lived in remarkably different habitats (iron-rich deep sea vent for PV-4 versus the iron-poor freshwater for MR-1). We have found that the RpoD-dependent HemH1 and RpoE2-dependent HemH2 might be complementary in cellular functions in PV-4 and MR-1. Here we also demonstrated that the two paralogues are functionally redundant despite such genetic divergence (Additional file 1: Figure S4). The differential expression of RpoE2-dependent *hemH2* gene in these two *Shewanella* species and the difference in their gene content, particularly the presence or loss of the gene cluster encoding the TonB-dependent heme iron sequestration and utilization system (*hmu*) and ABC-type hemin uptake system (loci SO_3668-SO_3675 in MR-1), were due to bacterial speciation and adaptation to the specific habitats in which different *Shewanella* species evolved. The iron-rich environment probably might have caused the loss of genes coding for iron-sequestration and relaxation of selective pressure on the maintenance of fully functional RpoE2-dependent HemH2 in PV-4. On the other hand, the multiple siderophore uptake systems (encoded by SO_1156, SO_3033, SO_3914 and SO_4516) might be crucial for the survival of MR-1 in the iron-limiting freshwater habitats. Therefore, the environmental pressure might have resulted in the complex evolution and speciation of different *Shewanella* species, in terms of not only gene gain and loss, but also differential expression of orthologous genes, for rapid adapt to the changing environments.

## Conclusion

Heme is a prosthetic group for a series of proteins including cytrochromes crucial for bacterial respiration, growth and stress response. Here we show that *S. oneidensis* MR-1 could uptake and utilize exogenous heme (hemin) for cytochrome biosynthesis and respiration while *S. loihica* PV-4 could not. Furthermore, bacterial genetic divergence may be attributed to differential gene expression of orthologous genes. The bacterial adaptation of PV-4 to the iron-rich deep-sea vent habitat is characterized by loss of iron- and heme-sequestration systems and loss of full functionality of *hemH2* paralogue. On the other hand, the transcription of *hemH1* and *hemH2* paralogues are well-coordinated for heme and cytochrome biosynthesis in the MR-1 strain living in iron-poor freshwater habitats since no PPIX was overproduced and released in the single mutants. In summary, bacterial evolution and speciation of *Shewanella* are characterized by not only different gene content but also differential expression of orthologous genes.

## Materials and methods

### Bacterial strains, plasmids, media, and culture conditions

*Shewanella lohica* PV-4 (ATCC® BAA-1088™) and *Shewanella oneidensis* MR-1(ATCC® 700550™) were cultured at 28 °C in Luria-Bertani (LB) broth/plates or the modified M1 minimum media (supplemented with 15 μg/ml of gentamycin or 50 μg/ml of kanamycin when necessary, and/or different levels of hemins (Sinopharm Chemical Reagent Co., Ltd., Beijing)). *Escherichia coli* strains were cultured at 37 °C in LB broth. The bacterial strains and plasmids used in this study are listed in Table 1. Since genetic manipulation of the wild-type PV-4 strain is difficult possibly due to the presence of a PstI restriction-modification system, we created a *ΔpstIΔpstM* double in-frame deletion mutant, which be used as a parental strain (here, PV-4 refers to the parental strain) for the subsequent tests [4]. Primers used in this study were listed in Table 2.

**Table 1:** Bacterial strains and plasmids

Strain	Description	Source or reference
*Escherichia coli* WM3064	*thrB1004 pro thi rpsL hsdS lacZDM15 RP4–1360 (araBAD)567 dapA1341::[erm pir(wt)]*	W. Metcalf
*E. coli* TOP10	*F*^*−*^ *mcrA* Δ(*mrr-hsdRMS-mcrBC*) Φ80*lacZ*DM15 Δ *lacX74 deoR recA1 araD139* Δ(*ara-leu*)*7697 galU galK rpsL* (Sm^r^) *endA1 nupG*	Invitrogen
*E. coli* EC100D+	*F*^*−*^ *mcrA Δ(mrr-hsdRMS-mcrBC) φ80dlacZΔM15 ΔlacX74 recA1 endA1 araD139 Δ(ara, leu)7697 galU galK λ- rpsL (Str*^*R*^*) nupG pir*^*+*^*(DHFR)*	Epicentre Technologies
*E. coli* DH5α	*F*^*−*^ *endA1 glnV44 thi-1 recA1 relA1 gyrA96 deoR nupG Φ80dlacZΔM15 Δ(lacZYA-argF)U169, hsdR17(r*_*K*_^*−*^ *m*_*K*_^*+*^*) phoA λ*–	Takara
*Shewanella oneidensis* MR-1	Dissimilatory metal-reducing strain isolated from Lake Oneida, New York	[40]
MR-1Δ*hemH1*	In-frame deletion mutant of *hemH1* gene (*SO_2019*) derived from MR-1	This study
MR-1Δ*hemH2*	In-frame deletion mutant of *hemH2* gene (*SO_3348*) derived from MR-1	This study
MR-1Δ*hemH1*Δ*hemH2*	In-frame deletion double mutant of *hemH1* (*SO_2019*) and *hemH2* (*SO_3348*) derived from MR-1	This study
*Shewanella* *loihica* PV-4	Iron-rich microbial mat at a hydrothermal vent of Loihi Seamount, Pacific Ocean	[41]
Modified *Shewanella* *loihica* PV-4	*pstI* (*Shew_0993*) and *pstM* (*Shew_0992*) double deletion double mutant derived from PV-4	This study
PV-4 ΔhemH1	In-frame deletion mutant of *hemH1* (*Shew_2229*) derived from the modified PV-4	This study
Plasmids		
pminiHmarRB1	mariner transposon delivery plasmid R6K, *Km*^*r*^	[3]
pDS3.0	Suicide vector derived from pCDV224; *Amp*^*r*^*, Gm*^*r*^*, sacB*	[42]
pDS3.0-PV-hemH1ko	Suicide plasmid for deletion of *hemH1* derived from PV-4	This study
pDS3.0-MR-hemH1ko	Suicide plasmid for deletion of *hemH1* derived from MR-1	This study
pDS3.0-MR-hemH2ko	Suicide plasmid for deletion of *hemH2* derived from MR-1	This study
pHERD30T	Shuttle vector with pBAD promoter, *Gm*^*r*^	[43]
pHERD30T-hemH1_slo_	pHERD30T containing the *hemH1* derived from PV-4, *Gm*^*r*^	This study
pHERD30T-hemH1_so_	pHERD30T containing the *hemH1* derived from MR-1, *Gm*^*r*^	This study
pHERD30T-hemH2_so_	pHERD30T containing the *hemH2* derived from MR-1, *Gm*^*r*^	This study
pET28a	Expression vector with T7lac promoter	Novogene
pET28a-katB	Overexpression construct of katB	This study
pET28a-cymA	Overexpression construct of cymA	This study

**Table 2:** Primers used in this study

Primer	Oligonucleotide sequence (5′-3′)
Complementation	
MR1-hemH1-F	5′- GTGAGCTCTATGCGACTGAATGTTAGCC − 3′
MR1-hemH1-R	5′- GCTCTAGATCATTCTGCACTTATCCAAG − 3′
MR1-HemH2-F	5′- GGAATTCAGAAATGGGTCACGCTGC − 3′
MR1-HemH2-R	5′- GCTCTAGAGCTTACAGATACGGCTTCACC − 3′
PV4-hemH1-F	5′- GGAATTCTAAAGTGAAGCGCTATATGAGC − 3′
PV4-hemH1-R	5′- GCTCTAGAGCAGTGATTTATGCCGTTTACC − 3’
pET28a-KatB-F	5′- GGAATTCATGAGTCAACAGTATTTAAC − 3’
pET28a-KatB-R	5′- GCAAGCTTTTATAACCCCAGCGCCATTTTG − 3’
pET28a-cymA-F	5′- GGAATTCATGAACTGGCGTGCACTATTT − 3’
pET28a-cymA-R	5′- GCAAGCTTTTATCCTTTTGGATAGGGGTG − 3’
Mutagenesis	
MR-hemH1_5O	5′- CAGAGCTCTCGTCGGCCAAAATAGAATA-3’
MR-hemH1_5I	5′- GACTGGCTTAGGTCGTCTCTCCAAGGCGTGAAAAGTGTCG-3’
MR-hemH1_3I	5′- AGAGACGACCTAAGCCAGTCGACGACCCGAGGATCACTTA-3’
MR-hemH1_3O	5′- GTGAGCTCGATGGCGGCAAACGGTATTA − 3’
MR-hemH2_5O	5′- CAGAGCTCGATGAACGCTAAAACCGTA − 3’
MR-hemH2_5I	5′- GACTGGCTTAGGTCGTCTCTCGCTCAATGATAATGATGA − 3’
MR-hemH2_3I	5′- AGAGACGACCTAAGCCAGTCGGATAGCTTAGCCGTTTGT − 3’
MR-hemH2_3O	5′- GTGAGCTC ACTGCGAGTAATTGTGGTT − 3’
PV4-hemH1_5O	5′- AGAGCTCGTACGTTCACCGCACTCTGA − 3’
PV4-hemH1_5I	5′- TGCATCGAGTTGATTGTCGCATCACTGCCCGATGCTATTT − 3’
PV4-hemH1_3I	5′- GCGACAATCAACTCGATGCATAGGCTAGCACCCACCCTTG − 3’
PV4-hemH1_3O	5′- AGAGCTCGTTAAAGCGGGAACGCCTCT − 3’
QRT-PCR	
QRT-PV4-hemH1-F	5′- CAGTGTCAACGTACTGCCGA − 3’
QRT-PV4-hemH1-R	5′- ATAAAGCTCTCCTTGCCGCC − 3’
QRT-PV4-hemH2-F	5′- ATTTCTGCATGCGTTACGGC − 3’
QRT-PV4-hemH2-R	5′- GCCAGCGCCGCAATATAATC − 3’
QRT-MR-hemH1-F	5′- CAACGGATTTAAGCGCCACCT − 3’
QRT-MR-hemH1-R	5′- CTGACCATGGGTTTTCCAATG − 3’
QRT-MR-hemH2-F	5′- GGTTTTACCGCTCTATCCGC − 3’
QRT-MR-hemH2-R	5′- CCGTATAGGGTTGCAACCAC − 3’
QRT-MR-rpoE2-F	5′- CGGGTACAGCATAACCGAGAAG − 3’
QRT-MR-rpoE2-R	5′- AATCATGTTGTGTCTCCAAAAAGC − 3’
QRT-MR-cymA-F	5′- CAAGTACAGATGCGTTCTG − 3’
QRT-MR-cymA-R	5′- GTAAGCCAATGCTTTGTCG − 3’
QRT-MR-napB-F	5′- GGATTAGGCTTAACGGCTACGGC − 3’
QRT-MR- napB -R	5′- TGTGGGTAAAAACATGGCGTG − 3’
QRT-MR-hmuX-F	5′- CCAGATTTAATGCCTGCACAA − 3’
QRT-MR-hmuX-R	5′- GCATGCCATCCAATTTAAGAT − 3’
QRT-MR-exbB-F	5′- GCATTCCTCGCCTTGATGAT − 3’
QRT-MR-exbB-R	5′- GCTTGCAGCTCCCTTTGTTG − 3’
QRT-MR-hmuC-F	5′- GATCGTTTATCGTCTGGCGAG − 3’
QRT-MR-hmuC-R	5′- GCTTCAGCTTCACCCAGTAGC − 3’
QRT-MR-hemA-F	5′- CACAGTTGCCTTAACCATTG − 3’
QRT-MR-hemA-R	5′- CTCACACATGCTTTGGGCAC − 3’
QRT-MR-hemB-F	5′- GCAACGCGCAGTGCGTGAAC − 3’
QRT-MR-hemB-R	5′- GTAGGCCATGATTTGAGTG − 3’
QRT-MR-hemD-F	5′- CTGACGATACGTTTAAAGCC − 3’
QRT-MR-hemD-R	5′- GCTTCACGGCCACCAACAC − 3’
QRT-MR-hemF-F	5′- CACCCATGCCAACGTGCGC − 3’
QRT-MR-hemF-R	5′- CTTTTATCGAAGCCTGCTTG − 3’
QRT-MR-hemG-F	5′- CAGATGTTGATAAGGTATTG − 3’
QRT-MR-hemG-R	5′- CAGATCATTGTGCGGTCGAA − 3’
QRT-16S-F	5′- TAATGGCTCACCAAGGCGAC − 3’
QRT-16S-R	5′- GGAGTTAGCCGGTCCTTCTTC − 3’
RT-PCR	
MR-hemH1-FTF	5′- CAACGGATTTAAGCGCCACCT − 3’
MR -hemH1-RTR	5′- CTGACCATGGGTTTTCCAATG − 3’
MR -hemH2-FTF	5′- GGTTTTACCGCTCTATCCGC − 3’
MR -hemH2-RTR	5′- CCGTATAGGGTTGCAACCAC − 3’
MR -rpoE2-FTF	5′- CGGGTACAGCATAACCGAGAAG − 3’
MR -rpoE2-RTR	5′- AATCATGTTGTGTCTCCAAAAAGC − 3’
16S rRNA-FT-F	5′-GTTGGAAACGACTGCTAATACC − 3’
16S rRNA-RT-R	5′-GGTCCTTCTTCTGTAGGTAACG − 3’

### Transposon mutagenesis, in-frame deletion and complementation

The mariner transposon mutant library (pmini*Hmar* RB1, courtesy of Daâd Saffarini, University of Wisconsin, Milwaukee, WI) preparation, mutant screening, and insertion site mapping were conducted as previously described [3]. The genetic manipulation was conducted in the wild type strain of MR-1, and the Δ*pstI*Δ*pstM* double mutant of PV-4. To generate the in-frame deletion mutants, the two-step protocol of selection (single cross-over, antibiotic resistance) and counter-selection (double crossover, sucrose sensitivity) was conducted using the suicide vector pDS3.0 (R6K replicon, *sacB*, Gm^R^)-based constructs with a fusion of upstream and downstream sequences of the target gene as previously described [42]. For genetic complementation, the target genes were PCR amplified and cloned into the pHERD30T or pBBR1MCS-2 vector [43, 44]. The resultant constructs and empty vector were transformed into the *Shewanella* wild type strains or mutant strains via conjugation using *Escherichia coli* WM3064 as a donor strain.

### RNA extraction, real-time PCR and RT-PCR analysis of gene transcription

Total RNA was extracted by using RNAiso Plus (Takara) and RNAprep pure Cell/Bacteria Kit (TIANGEN BIOTECH (Beijing) CO., LTD.). To prepare cDNA, 2 μg of total RNA was reverse transcribed using the PrimeScript® RT reagent Kit with gDNA Eraser (Takara) according to the manufacturer’s protocol. Semi-quantitative PCR analyses were carried out as described previously [4, 30]. Quantitative real-time PCR was performed as previously described [4, 30]. The primers used are listed in Table 2.

### Extraction, chemical and spectral analyses of the extracellular compound

Cells from 100 ml cultures were pelleted by centrifugation. The pellet was subjected to a rapid wash with pure ice-cold water and centrifuged. After the process was repeated, the pellet was incubated with 4 ml of a 9:1 mixture of acetone-0.1 N NH_4_OH for 1 h at 4 °C. Cell debris was removed by centrifugation at 13000 g and 4 °C for 15 min, and the supernatant was analyzed for PPIX by mass spectrometry. The ultraviolet-visible spectrograms were plotted as previously described [4, 30]. Electrospray ionization tandem mass spectrometry (ESI-MS/MS) analyses were conducted by using Finnigan LCQ Advantage Max Mass Spectrometer (Thermo Finnigan).

### Nitrate reduction, H_2_O_2_ and light sensitivity test

Nitrate reduction was examined in MR-1 and mutants cultured in modified M1 medium with sodium nitrate as the electron acceptor. The cultures were incubated under anaerobic or microoxic conditions (without shaking). Nitrate and nitrite concentrations were measured with a standard colorimetric assay [45]. To test the bacterial resistance to hydrogen peroxide, wild type and mutant cells were grown overnight in LB broth containing different levels of hydrogen peroxide (0, 0.1, 0.3, 0.5, 0.7 and 1 mM) and growth was monitored by measuring optical density at 600 nm as previously described [46]. Cell patches grown from a droplet of mid-log-phase culture (OD_600_ of ~ 0.2) for each indicated strain on LB plates (supplemented with 10 μg/ml hemin and 15 μg /ml gentamycin). All samples were examined under dark and visible light (about 700–1,000 lx).

### KatB peroxidase activity assay

Cells were washed twice in 20 ml cold 0.05 KPi (pH 7.8), resuspended in 1 ml 0.01 M KPi (pH 6.4) and lysed by sonication. Cell debris was removed by centrifuging at 12000 g at 4 °C for 10 min, and the supernatants were used to assay the KatB activity by a widely used protocol with some modifications [47]. 3 ml of the supernatants was mixed with 3 ml of phosphate buffer containing 0.1 M hydrogen peroxide. The reaction was stopped by adding 3 ml of 10% (v/v) sulfuric acid and the residual hydrogen peroxide was titrated against 0.1 M permanganate (KMnO_4_) solution until a faint purple color persisted for at least 30 s. The total protein concentrations were measured by using a total protein assay kit (Jiancheng Biotech., Nanjing, China). The same amounts of boiling-denatured supernatants were used as control.

### SDS-PAGE electrophrosis and heme staining

The harvested *Shewanella* cells were homogenized by applying high pressures (JN-02C low temperature ultra-high pressure continuous flow cell disrupter, Juneng Biol. and Technol. Co., Guangzhou, China) or Ultrasonic Cell Disruption System (SCIENTZ-IID, Ningbo Xingzhi Biotechnology Co., China) and centrifuged at 4 °C. The supernatants containing the cellular protein fraction were resuspended in the SDS loading buffer and separated by SDS-PAGE using 12% polyacrylamide gels. Heme stains were performed using 3, 3′, 5, 5′-tetramethyl benzidine dihydrochloride as previously described [48]. Images were visualized with the Gel Doc™ XR+ system (Bio-Rad Laboratories, Inc. United Kingdom).

### Western blot analysis

Subcultures were grown in LB with 50 μg kanamycin ml^− 1^ at 28 °C and 220 rpm for 8 h. The protein samples were then prepared by the standard procedures of the KeyGEN Biotech kit (KGP450, keyGEN). The protein concentrations of the lysates were measured by a total protein assay kit (Jiancheng Biotech., Nanjing, China). Proteins were analysed by 12% SDS-PAGE and electro-transferred onto the 0.45 μm PVDF membrane in transfer buffer (47.8 mM Tris, 36.7 mM glycine, 1.3 mM SDS, 20% (v/v) methanol). His-tagged proteins were probed with His monoclonal primary antibodies (Beyotime) at 1:1000 dilution and immuno-coupled with anti-Mouse IgG (H + L)-HRP (Beyotime) according to the manufacturer’s instructions. ECL Plus (Biosharp) was used for detection, and film images were digitized using ImageQuant LAS4000mini.

### Bioinformatics tools

Nucleotide sequences of genes were retrieved from KEGG (Kyoto Encyclopedia of Genes and Genomes) or NCBI database by using BLAST searches at the National Center for Biotechnology Information (http://www.ncbi.nlm.nih.gov/BLAST). The ClustalW2 package (http://www.ebi.ac.uk/Tools/msa/clustalw2/) was used for nucleotide sequence alignments.

## Additional file

Additional file 1: **Tables S1-S2** and **Figures S1-S4** associated with this manuscript. (DOCX 1418 kb)

## Data Availability

All data generated or analyzed during this study are included in this published article and its supplementary information files.
