# Supplementary material for: Differential gene content and gene expression for bacterial evolution and speciation of Shewanella in terms of biosynthesis of heme and heme-requiring proteins
Source: BMC Microbiol. 2019 Jul 30;19:173. doi: 10.1186/s12866-019-1549-9 (PMC6664582; doi:10.1186/s12866-019-1549-9)
Supplement: Supplementary file 1 — Tables S1-S2 and Figures S1-S4 associated with this manuscript. (DOCX 1418 kb) [file 12866_2019_1549_MOESM1_ESM.docx]

**Additional file 1**

**Journal: BMC Microbiology**

**Differential gene content and gene expression for bacterial evolution and speciation of Shewanella in terms of biosynthesis of heme and heme-requiring proteins**

Jingcheng Dai^1^, Yaqi Liu^1^, Shuangyuan Liu^1^,Shuyang Li^1^, Na Gao^1^, Jing Wang^1^, Jizhong Zhou^2,3^, Dongru Qiu^1*^

^1^ Institute of Hydrobiology, Chinese Academy of Sciences, Wuhan 430072, China and University of Chinese Academy of Sciences, Beijing 100049, China

^2^ Institute for Environmental Genomics, and Department of Microbiology and Plant Biology, University of Oklahoma, Norman, OK 73019, USA

^3^ Earth Science Division, Lawrence Berkeley National Laboratory, Berkeley, CA 94270, USA

**^*^Correspondence to: Dongru Qiu, PhD**

Institute of Hydrobiology, Chinese Academy of Sciences

Wuhan 430072, Hubei Province, China

Phone: 0086-27-68780215; Fax: 0086-27-68780123

E-mail: qiu@ihb.ac.cn

**Table S1** The heme proteins of *S. oneidensis* MR-1.

| **ID** | **Gene** | **Protein** | **Biological process** | **Prostetic group** |
| --- | --- | --- | --- | --- |
| SO_1070 | *katB* | Catalase B | H_2_O_2_ scavenging | Protoheme IX, heme D? |
| SO_4405 | *katG1* | Catalase G |  | Protoheme IX, heme B |
| SO_0725 | *katG2* | Catalase G |  | Protoheme IX, heme B |
| SO_1776-SO_1778 | *mtrABC* | Extracellular iron oxide respiratory  protein | Electron transfer | Protoheme IX, heme B |
| SO_1779 | *omcA* |  |  | Protoheme IX, heme B |
| SO_1780-SO_1782 | *mtrDEF* | Extracellular respiratory  protein |  | Protoheme IX, heme B |
| SO_3285-SO_3286 | *cydAB* | Cytochrome bd oxidase-II |  | Protoheme IX, (heme D?) |
| SO_1427-SO_1428 | *dmsEF* | Periplasmic decaheme cytochrome c |  | Protoheme IX, heme B |
| SO_4606-SO_4607 | *coxBA* | Cytochrome C oxidase |  | Protoheme IX, heme A |
| SO_4609 | *coxC* | Cytochrome C oxidase |  | Protoheme IX, heme A |
| SO_4613-SO_4614 | *ctaA-ctaB* | Cytochrome C oxidase |  | Protoheme IX, heme A |
| SO_0790 | *fccA* | Fumarate reductase flavoprotein |  | Protoheme IX, heme B? |
| SO_2361-SO_2364 | *ccoPQON* | Cytochrome C oxidase |  | Protoheme IX, heme A |
| SO_0101-SO_0103 | *fdnGHI* | Formate dehydrogenase-O |  | Protoheme IX, heme B/D |
| SO_1233 | *torC* | Trimethylamine-N-oxide reductase |  | Protoheme IX, heme B |
| SO_4591 | *cymA* | Tetraheme cytochrome c |  | Protoheme IX, heme B |
| SO_1927-SO_1930 | *sdhCDAB* | Succinate dehydrogenase | Electron transfer, TCA cycle | Protoheme IX, heme B |
| SO_1111-SO_1112 | *bfr* | Bacterioferritin | Iron storage | Protoheme IX, heme B |
| SO_2178 | *ccpA* | Cytochrome C peroxidase | Unknow | Protoheme IX, heme B |
| SO_0740 | *unknow* | Dyp-type heme-dependent peroxidase | Unknow | Protoheme IX, heme B |
| SO_3980 | *nrfA* | Nitrite reductase | Anaerobic respiration | *Siroheme |
| SO_4568 | *nrfD* |  |  |  |
| SO_0845-SO_0849 | *napBHGAD* | Periplasmic nitrate reductase | Anaerobic respiration | Protoheme IX, heme B |
| SO_1783-SO_1784 | *feoAB* | Ferrous iron transporter | Uptake of ferrous iron under conditions of iron limitation and low pH | Protoheme IX, heme B |
| SO_3737-SO_3738 | *cysIJ* | Sulfite reductase | Sulfate assimilation | *Siroheme |

*Siroheme synthesis departs from the pathway of heme synthesis after the HemD step and does not involve coproporphyrinogen III oxidase (HemN/HemF) or ferrochelatase (HemH).

**Table S2** Iron acquisition and metabolism function between PV-4 and MR-1.

| Strains | Function | Expressed protein |
| --- | --- | --- |
| MR-1 | Siderophore assembly kit | Siderophore biosynthesis protein, monooxygenase |
| MR-1 | Siderophore assembly kit | Siderophore synthetase component, ligase |
| MR-1 | Siderophore assembly kit | Siderophore synthetase small component, acetyltransferase |
| MR-1 | Heme, hemin uptake and utilization systems in Gram-Negatives | ABC-type hemin transport system, ATPase component |
| MR-1 | Heme, hemin uptake and utilization systems in Gram-Negatives | Biopolymer transport protein ExbD1 |
| MR-1 | Heme, hemin uptake and utilization systems in Gram-Negatives | Hemin ABC transporter, permease protein |
| MR-1 | Heme, hemin uptake and utilization systems in Gram-Negatives | Periplasmic hemin-binding protein |
| MR-1 | Heme, hemin uptake and utilization systems in Gram-Negatives | Putative heme iron utilization protein |
| MR-1 | Heme, hemin uptake and utilization systems in Gram-Negatives | phosphate oxidase-related putative heme iron utilization protein |
| MR-1 | Heme, hemin uptake and utilization systems in Gram-Negatives | TonB-dependent hemin , ferrichrome receptor |
| MR-1 | Heme, hemin uptake and utilization systems in Gram-Negatives | Transcriptional regulator LuxT |
| MR-1 | Transport of Iron | Iron-uptake factor PiuC |
| PV-4 | Siderophore Aerobactin | Aerobactin siderophore receptor IutA |
| PV-4 | ABC transporter [iron.B12.siderophore.hemin] | ABC transporter (iron.B12.siderophore.hemin) , ATP-binding component |
| PV-4 | Transport of Iron | Iron-regulated protein A precursor |
| PV-4 | Transport of Iron | Iron-uptake factor PiuB |
| PV-4 and MR-1 | Encapsulating protein for DyP-type peroxidase and ferritin-like protein oligomers | Predicted dye-decolorizing peroxidase (DyP), YfeX-like subgroup |
| PV-4 and MR-1 | Hemin transport system | Ferric siderophore transport system, periplasmic binding protein TonB |
| PV-4 and MR-1 | Hemin transport system | Outer membrane receptor proteins, mostly Fe transport |
| PV-4 and MR-1 | Iron acquisition in Streptococcus | Ferric iron ABC transporter, ATP-binding protein |
| PV-4 and MR-1 | Iron acquisition in Streptococcus | Ferric iron ABC transporter, iron-binding protein |
| PV-4 and MR-1 | Iron acquisition in Streptococcus | Ferric iron ABC transporter, permease protein |
| PV-4 and MR-1 | Transport of Iron | Ferric iron ABC transporter, ATP-binding protein |
| PV-4 and MR-1 | Transport of Iron | Ferric iron ABC transporter, iron-binding protein |
| PV-4 and MR-1 | Transport of Iron | Ferric iron ABC transporter, permease protein |
| PV-4 and MR-1 | Transport of Iron | Ferric uptake regulation protein FUR |
| PV-4 and MR-1 | Transport of Iron | Ferrichrome-iron receptor |
| PV-4 and MR-1 | Transport of Iron | Ferrous iron transport protein A |
| PV-4 and MR-1 | Transport of Iron | Ferrous iron transport protein B |

**Figure S1** the double deletion of *hemH1* and *hemH2* in MR-1 strain


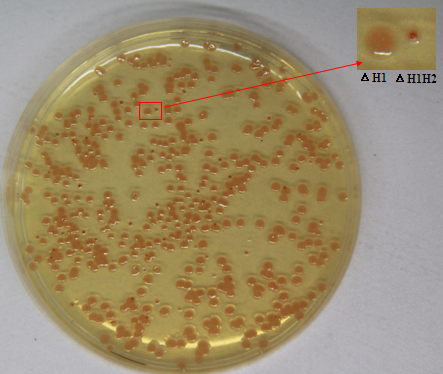

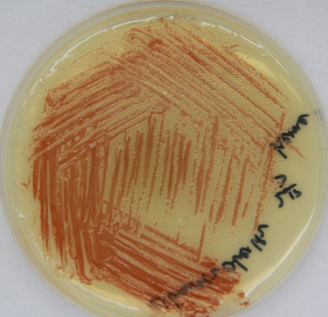


**Figure S2** Ultraviolet-visible spectrograms of the bacterial extract (the wildtype and the *hemH1* mutant). Absorbance was measured at every 5 or 10 nanometers (nm).

**Figure S3** Alignment of promoter sequences upstream of loci SO_3349-SO_3348 (*pgpD-hemH2*) of *S. oneidensis* MR-1 and Shew_1140 (*hemH2*) in *S. loihica* PV-4. The putative RpoE2 recognizing motifs (-35 motif TGATCC/T and -10 motif CGTAA/CT) are highlighted in yellow.

CLUSTAL 2.1 multiple sequence alignment

SO_3349 TCCTATTTTGTCATATGTTAAAGCAGCTTTTTGAAGCTGCTTTTTATCGCTTAGGGTAAT 60

SHEW_1140 -CC--TCTTATTGCACTTTGAAAAAACC--------------------------GATAAT 31

** * ** * * ** ** * * * ****

SO_3349 AGATAACTCCTTGTAGCTTTTATTTTTTTATT-CAATATGATCCCTATCGTAGCAAGTTA 119

SHEW_1140 ------CCCTTTATCGGTTTT-TTTATGTGTGGCGAT-TGATCTCTAAGGGGCCGAACCG 83

* * ** * * **** *** * * * * ** ***** *** * * *

SO_3349 CGTAATAAGAATAAGTTCGCCTATCTTGGATCATTGAA-TGAGAAGGACATT--- 170

SHEW_1140 CGTACTGA---------TGCCCA---TGCATCATTAGCCTCGGCCAGGTGCCCAG 126

**** * * *** * ** ****** * * *

**Figure S4** Distance tree of *hemH* paralogues in *Shewanella* based on DNA sequence similarity. *E.coli hemH* used as reference. Clearly, the two *hemH* paralogues fall into two cluster, *hemH1* sequences form the upper gene cluster, while *hemH2* sequences form the lower cluster.


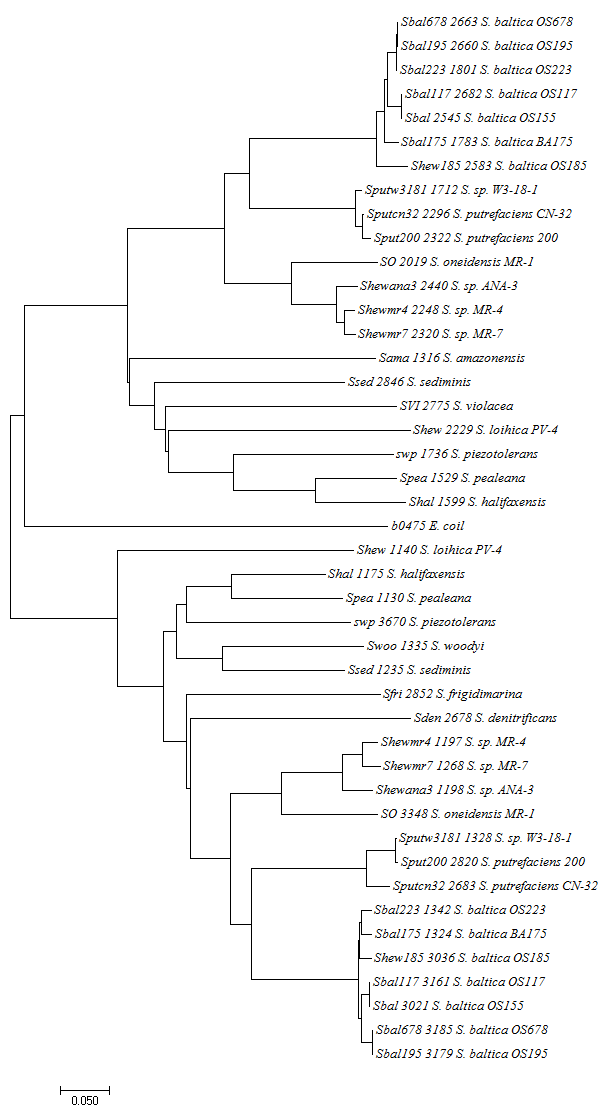


*hemH1*

*hemH2*
